# In Vivo Confocal Microscopy and Anterior Segment Optical Coherence Tomography in Optimizing Diagnosis and Therapeutic Management in Fungal Keratitis: Case Reports and Literature Review

**DOI:** 10.3390/jcm14228066

**Published:** 2025-11-14

**Authors:** Alina Gabriela Gheorghe, Ana Maria Arghirescu, Maria Cristina Marinescu, Ancuța Georgiana Onofrei, Doina Mihaela Pop, Liliana Mary Voinea, Radu Constantin Ciuluvică

**Affiliations:** 1 Department of Ophthalmology, Clinical Institute of Ophthalmological Emergencies “Prof. Dr. Mircea Olteanu”, 010464 Bucharest, Romania; alina.gheorghe.g@gmail.com (A.G.G.);; 2 Department of Ophthalmology, Carol Davila University of Medicine and Pharmacy, 020021 Bucharest, Romania; 3 Doctoral School, Carol Davila University of Medicine and Pharmacy, 020021 Bucharest, Romania; 4 Medical Physiology Discipline, Carol Davila University of Medicine and Pharmacy, 020021 Bucharest, Romania; 5 Foisor Orthopaedic Hospital, 030167 Bucharest, Romania; 6 Department of Anatomy, Faculty of Dental Medicine, Carol Davila University of Medicine and Pharmacy, 020021 Bucharest, Romania

**Keywords:** fungal keratitis, in vivo confocal microscopy, anterior segment OCT, deep anterior lamellar keratoplasty, infectious keratitis, acanthamoeba keratitis, coinfection, therapeutic keratoplasty, penetrating keratoplasty, in vivo histology, candida keratitis, fusarium keratitis

## Abstract

**Background**: Fungal keratitis remains a serious threat to vision, often progressing despite medical therapy and requiring surgical intervention. Therapeutic deep anterior lamellar keratoplasty (DALK) and therapeutic penetrating keratoplasty (TPK), are frequently required but carry risks of infection recurrence and graft rejection. As timely identification of the etiological agent is essential for improving the outcomes in infectious keratitis, in vivo confocal microscopy (IVCM) and anterior segment optical coherence tomography (AS-OCT) are instrumental in providing insights that can guide better therapeutic decision-making and improving outcomes in fungal keratitis. **Case Description**: We report the cases of two previously healthy patients (case one, 56-year-old woman; case two, 38-year-old man), who have presented in our service with unilateral infectious keratitis after ocular trauma with vegetable matter during outdoor activities, with a visual acuity of counting fingers and hand motion, respectively. Slit-lamp examination revealed unilateral extensive corneal infiltrates suggestive for fungal etiology in both cases. In vivo confocal microscopy (HRT-3, Heidelberg Retina Tomograph 3/Rostock Cornea Module, Heidelberg Engineering, Heidelberg, Germany) identified lesions suggestive for Candida Albicans and Acanthamoeba coinfection in case one and filamentous fungal keratitis in case two. Anterior segment optical coherence tomography (MS-39, CSO, Italy) was used to monitor the extent and morphology of the infiltrates. The patients underwent therapeutic DALK and TPK, respectively, with good results at the one-year follow-up. **Conclusions**: Our cases illustrate the advantages of incorporating IVCM and AS-OCT as complementary imaging techniques into clinical practice. IVCM and AS-OCT in fungal keratitis could lead to an earlier diagnosis, more accurate dynamic treatment response evaluation, and the identification of high-risk features for aggressive fungi for a more tailored medical and surgical management.

## 1. Introduction

Fungal keratitis represents a sight-threatening infection with high prevalence and incidence, especially in tropical and subtropical regions. The causative species can be classified as yeasts (e.g., Candida) or filamentous fungi (either pigmented—such as Alternaria—or nonpigmented—such as Fusarium, Aspergillus) [1,2]. Studies at Moorfield Eye Hospital and in the United States report filamentous fungi as the main agent in over 69% of documented cases [3,4], yeasts in 25% [3] and 20% [4], and other non-filamentous or unidentified fungi in the remaining 6%, respectively [4]. Bacterial coinfection was found in 17.9% of the cases [3].

In vivo confocal microscopy (IVCM) can provide near-histological, noninvasive “in vivo histopathology” of fungal keratitis, allowing for the dynamic monitoring of tissue architecture, inflammatory response, and pathogen morphology, enabling the integration of high-resolution imaging into both diagnosis and surgical decision-making. Anterior segment optical coherence tomography (AS-OCT) provides great utility in evaluating the extent, degree of involvement of the different corneal layers, and morphology of the infiltrate [5]. IVCM in particular has demonstrated high sensitivity and specificity for detecting fungal agents in infectious keratitis [6,7].

The purpose of this study was to illustrate the challenges of managing two aggressive infectious keratitis cases with a focus on innovative imaging techniques, as well as provide a concise literature review of the most common IVCM and AS-OCT characteristics of the most common fungal etiological agents.

## 2. Case Reports

This study was conducted in accordance with the Declaration of Helsinki and its subsequent amendments. Ethical approval was obtained from the Ethics Committee of the Clinical Institute of Ophthalmological Emergencies, “Prof. Dr. Mircea Olteanu” (3391/12 August 2025). Written informed consent was obtained prior to the intervention.

### 2.1. Case 1—Candida Keratitis with Acanthamoeba Coinfection

The following case illustrates how IVCM (HRT-3, Heidelberg Retina Tomograph 3/Rostock Cornea Module, Heidelberg Engineering, Heidelberg, Germany) findings completed the clinical picture, giving real-time information about the pathogen’s morphology and response to medical and surgical treatment.

**Figure 1:**
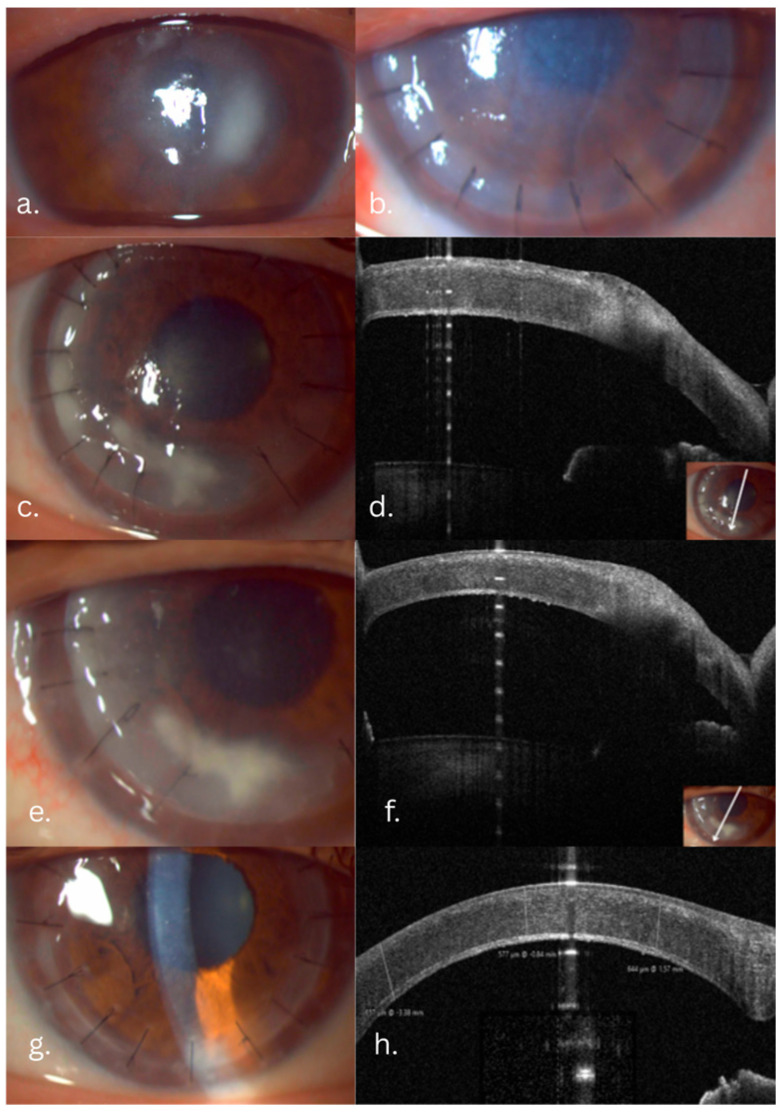
Candida fungal keratitis case evolution: slit-lamp and AS-OCT aspects. (**a**) Preoperative infiltrate: intact epithelium with stromal infiltrate, indistinct margins. (**b**) One day postoperative. (**c**) Five-month follow-up: white-gray infiltrate involving the graft–host interface and margins. (**d**) AS-OCT slice showing the extent of the infiltrate. (**e**,**f**) Slit-lamp and AS-OCT aspects after Voriconazole graft–host interface wash. (**g**,**h**) One-year follow-up, slit-lamp and AS-OCT aspects: transparent cornea, clear graft–host interface.

A 56-year-old agricultural worker sustained a corneal injury from a vine twig. At the presentation, visual acuity was counting fingers at 5 m. Slit-lamp examination revealed a central, anterior stromal infiltrate, with marked elevation in the nasal quadrant, with intact overlying epithelium and mild perilesional inflammation. AS-OCT (MS-39, CSO, Italy) confirmed infiltrate involvement limited to the anterior two-thirds of the stroma without endothelial involvement. Initial treatment involved antifungal treatment with systemic fluconazole (200 mg/day) and topical fluconazole (0.2% solution q2h). After one week, the patient showed no improvement. Candida Albicans was identified in fungal cultures several days after admission. Therapeutic deep anterior lamellar keratoplasty (DALK) was therefore performed. Postoperatively, AS-OCT demonstrated good graft–host integration and clear interfaces. Postoperative medical treatment consisted of topical fluconazole (0.2% solution q2h), Levofloxacin (0.5%: q.i.d.), and preservative-free artificial tears. Rejection prophylaxis was performed with topical cyclosporine (CsA) (1 mg/mL q12h). To lower the risk of recurrence of the fungal infection, topical corticosteroids were avoided. At the four-month follow-up, the graft remained clear, with no signs of immune rejection. Slit-lamp and AS-OCT evolution are illustrated in Figure 1. 

**Figure 2:**
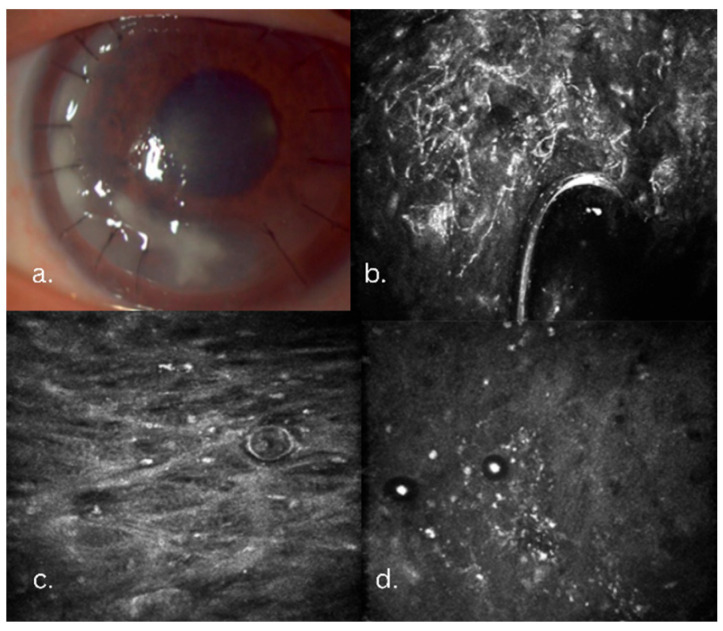
Five-month follow-up aspects, immediately after admission. (**a**) Infiltrate involving the graft–host interface; IVCM slices. (**b**) Linear and round hyperreflective bodies representing pseudohyphae and spores adjacent to a suture. (**c**,**d**) Stromal slices with round hyperreflective body representing Acanthamoeba cysts and trophozoite confirming fungal and Acathamoeba coinfection.

**Figure 3:**
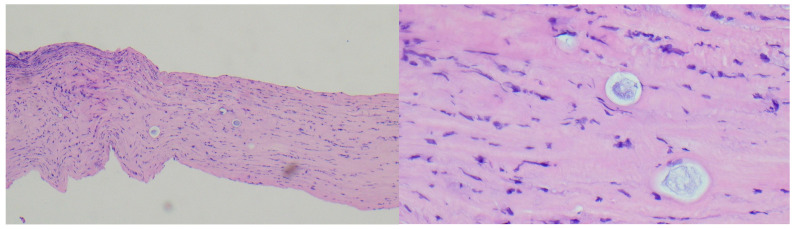
Histopathology slice showing Acathamoeba cysts and trophozoites at different depths in the corneal stroma.

**Figure 4:**
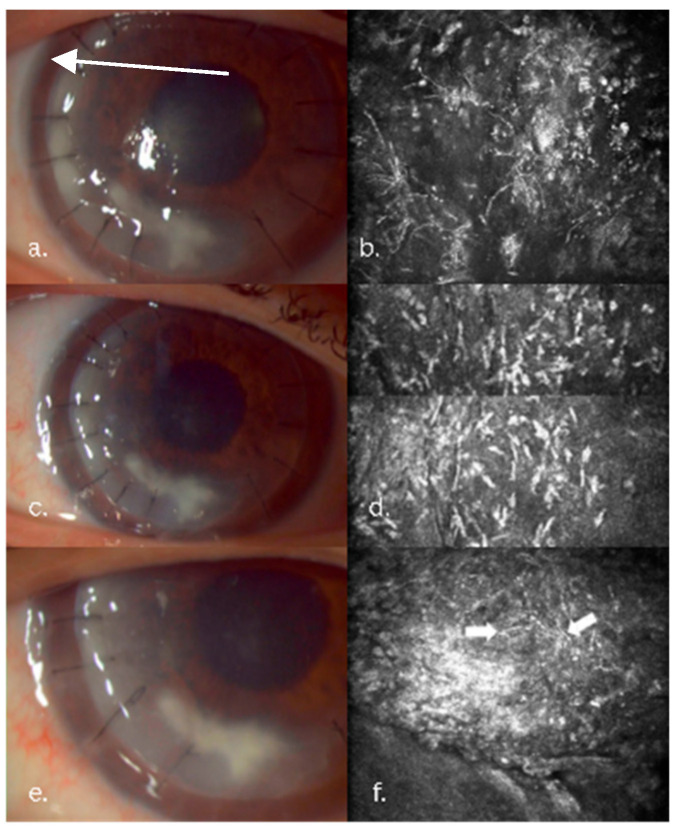
Clinical course in the Candida keratitis case. (**a**,**b**) At admission, slit-lamp and IVCM aspects: long, branched linear hyperreflective structures; pseudohyphal lesions, round hyperreflective bodies; spores. (**c**,**d**) At 3 days post-graft–host interface wash with Voriconazole. (**e**,**f**) At 10 days post graft–host interface wash.

At the five-month follow-up, the patient presented with decreased vision, and slit-lamp revealed new infiltrates involving the graft margins and the graft–host interface. IVCM showed fungal pseudohyphae, spores, Acanthamoeba cysts, and trophozoites. The Acanthamoeba lesions were confined to the anterior and mid-stroma. The fungal–Acanthamoeba coinfection was confirmed by IVCM in the anterior and mid-stroma (Figure 2). Histopathology confirmed the IVCM findings (Figure 3). Antifungal treatment included systemic voriconazole (200 mg q12h) and topical voriconazole (10 mg/mL q1h). Anti-Acanthamoeba treatment included topical povidone-iodine 2% (20 mg/mL q4h). Due to the extensive depth of the infiltrate and to avoid the extensive involvement of the host’s posterior stroma and endothelium, the DALK graft was lifted, infected sutures replaced, and an interface wash with voriconazole (50 µg/0.1 mL) was performed. Ten days post-wash, despite a mild clinical improvement, recurrence of the pseudohyphal network was confirmed by IVCM (Figure 4). Cultures subsequently revealed Candida albicans resistant to fluconazole and amphotericin, intermediately resistant to voriconazole, and sensitive to ketoconazole. Due to the persistent infection that was still confined to the original DALK graft, a second therapeutic DALK was successfully performed. Postoperative AS-OCT and IVCM showed excellent graft integration and the persistent absence of fungal or Acanthamoeba lesions, and maintained corneal clarity both in the early postoperative period, as well as at the two-year follow-up. Visual acuity improved progressively, reaching a BCVA of 0.6 at the three-month follow-up, which also remained stable at the two-year follow-up. Postoperative medical treatment consisted of topical ketoconazole 1% (10 mg/mL qid), CsA (1 mg/mL q12h), povidone-iodine 2% (20 mg/mL q6h), Levofloxacin (0.5%: q.i.d.), and lubricants.

### 2.2. Case 2—Filamentous Fungal Keratitis

The second case report refers to a 38-year-old male who presented to our Emergency service with a red, painful eye, decreased visual acuity—hand motion—and a fulminant, full-thickness central stromal infiltrate featuring fluffy margins, an overlying epithelial defect, endothelial plaque, hypopyon, and an organized anterior chamber membrane. Two months earlier he suffered a penetrating ocular trauma with corneal puncture and traumatic cataract due to contact with a rose thorn, managed surgically in a different center with lensectomy–vitrectomy.

**Figure 5:**
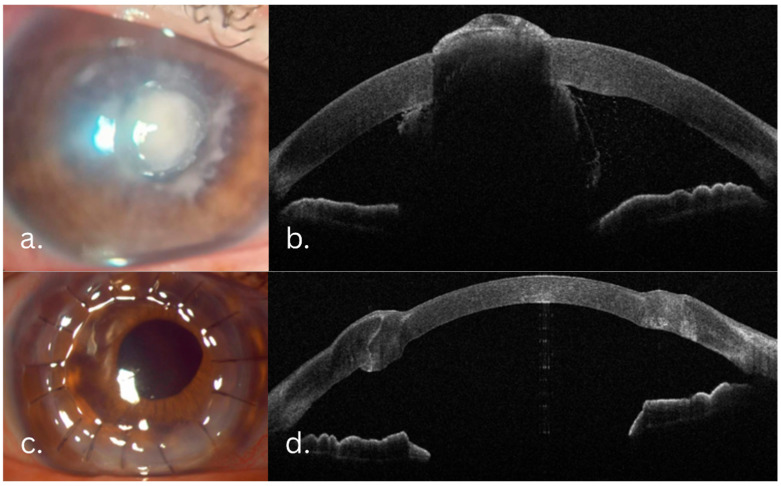
(**a**,**b**) Preoperative aspects—central stromal infiltrate featuring fluffy margins, an overlying epithelial defect, endothelial plaque, hypopyon. (**c**,**d**) Two months postoperation—well integrated, clear corneal graft.

**Figure 6:**
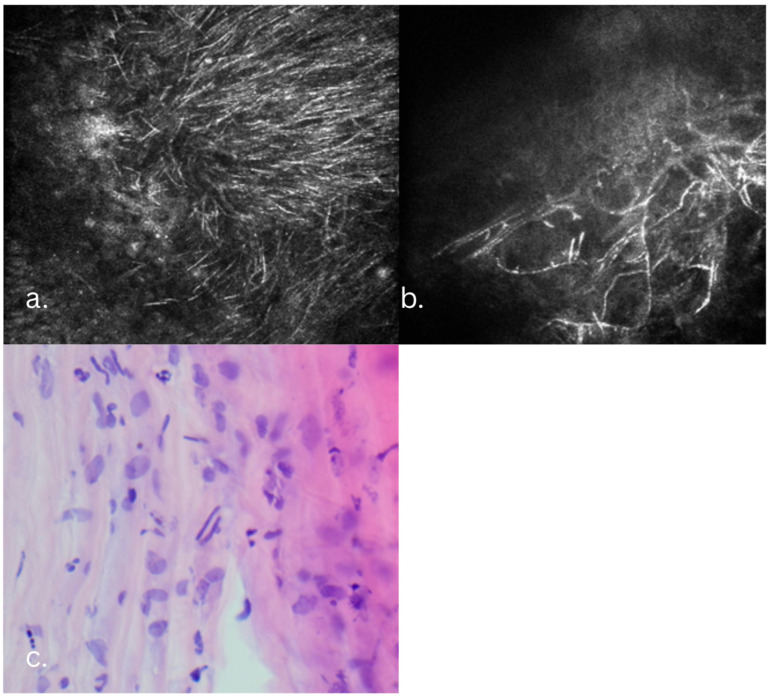
Preoperative IVCM slices and histopathology slice: (**a**) lesion edges, dense network of linear hyperreflective fungal hyphae; (**b**) long, branched fungal hyphae network—several budding branches can be seen; (**c**) histopathology slice—inflammatory cells interspersed with thin, long bodies suggestive of fungal lesions.

AS-OCT revealed an intensely hyperreflective, full-thickness stromal lesion with posterior shadowing, indicating deep stromal invasion (Figure 5). The endothelial plaque and anterior chamber membrane could also be observed. IVCM showed a dense network of long, linear, hyperreflective filaments interspersed with round bodies suggestive of spores, confirming active filamentous fungal infection (Figure 6). Mode B echography revealed hyperechogenic echoes in the vitreous, suggestive for inflammatory infiltrates. Culture results were available after several days, positive for *Fusarium* spp.

Medical treatment included systemic voriconazole (200 mg q12h), topical voriconazole (10 mg/mL q1h), topical cefuroxime (50 mg/mL q1h), gentamicin (14–15 mg/mL q1h), and artificial tears.

The full-thickness stromal infiltrate continued to extend, threatening to involve the posterior pole in the unicameral eye (due to previous lensectomy), despite being under local and systemic treatment; the IVCM and AS-OCT aspects and the high risk of perforation, all risk factors for poor prognosis, led to the patient undergoing an emergency TPK.

The postoperative course was performed with topical voriconazole (10 mg/mL q6h) and oral voriconazole (200 mg q12h for one month postoperation), topical levofloxacin (5 mg/mL q6h), povidone-iodine 2% (20 mg/mL q6h), and artificial tears. Rejection prophylaxis was performed with topical cyclosporine (CsA) (1 mg/mL q12h).

Two months after the TPK, BCVA was 0.2, and slit-lamp and AS-OCT confirmed a well-integrated, transparent graft without infiltrates. BCVA and corneal transparency were maintained at the one year follow-up.

## 3. Discussions and Literature Review

### 3.1. Clinical Diagnosis

Important risk factors for fungal keratitis, particularly filamentous, include ocular trauma with plant materials [8] and agricultural activities [9,10].

Candida cases are more often associated with preexisting corneal risk factors—contact lens wear [9], prolonged topical corticosteroid use [10] (which decreases corneal immune defense and increases the virulence of the fungi [11]), ocular surface disease (keratoconjunctivitis sicca, blepharitis, keratopathy) [3,7], or general immunosuppression [10].

The usual slit-lamp signs found are stromal infiltrates with indistinct margins, overlying epithelial defects, satellite lesions, and endothelial involvement [7]. A pigmented corneal ulcer is an important, if rare, indication of the fungal etiology [12]. Other characteristics may include the following: a raised infiltrate, a gray, ulcerated corneal epithelium, no epithelial defect over a stromal lesion which extends towards the endothelium, anterior chamber involvement (hypopyon), or a sterile circular stromal infiltrate (Wessely ring) [3]. Filamentous fungi are more likely to lead to a dry-looking, gray infiltrate, with indistinct, feathery edges, representative of hyphal growth, while Candida usually leads to a white, raised infiltrate, in an eye with preexisting ocular surface disease [11]. Candida can be distinguished from filamentous fungi from the lesion morphology: the borders are more defined, sometimes similar to a bacterial ulcer. Feathery branches are absent in Candida keratitis. Sometimes an epithelial plaque/raised lesion can be observed. It can form endothelial plaques [13]. In transplanted patients, on the graft, a Candida infection can present as infectious crystalline keratopathy, exhibiting branch-like stromal opacities [14].

### 3.2. Microbiological Examination

Identifying the causative organism in fungal keratitis is critical, but microbiological results are often delayed. Samples from the ulcer base and edges should be collected for microscopy and culture; if scraping is inconclusive but clinical signs strongly indicate fungal infection, corneal biopsy with diagnostic keratectomy is recommended [15]. Cultures are negative in 41–75% of clinically suspected fungal cases [16], making microscopic examination the gold standard [17].

### 3.3. In Vivo Confocal Microscopy

IVCM provides unrivaled detail resolution, being able to examine details as small as 1.5–4 μm. IVCM has shown high sensitivity for detecting fungal elements, often exceeding that of cultures [6,18].

The key characteristics of filamentous (*Aspergillus* spp., *Fusarium* spp.)- and yeast (*Candida* spp.)-type fungi are essential to differentiate, as the filamentous fungi carry a greater risk of perforation, due to their tendency to advance towards the posterior stroma and breach the Descemet’s membrane [19]. An overview of the most common IVCM findings is illustrated in Table 1. 

*Candida* spp. have a dimorphic nature; therefore, findings usually include multiple types of structure (spores, pseudohyphae, and, in the late phases of the infection, hyphae). Spores appear as round structures, 2–5 μm in diameter. Pseudohyphae (aggregated fungal cells) appear as elongated, matchstick-like structures (10–40 μm long, 5–10 μm wide). In Candida infections, usually a higher density of dendritic and inflammatory cells is present, both perilesional and interspersed with the fungal structures [2]. In terms of branching, Filamentous fungi, when developing the hyphal network, have a higher degree of dichotomization compared to yeasts [20]. A report on Candida parapsilosis by Scotto et al. described the pattern of spores as a caviar-like disposition of round, hyperreflective structures [21]. This type of Candida does not form hyphae [22].

Differentiation between filamentous fungi, particularly between *Fusarium* spp. and *Aspergillus* spp. based on the branching angles has been extensively analyzed, with Brasnu et al. concluding in 2006 that *Fusarium* spp. tend to branch at 90 degrees [2], while *Aspergillus* spp. tend to branch at 45 degrees. However, further studies showed no difference in branching angles between the two [22,23].

*Fusarium* spp. are characterized by brightly reflective, linear, branching, thin structures corresponding to hyphae (200–300 μm long and 3–5 μm wide). The septated, slightly beaded aspect of the hyphae can sometimes be identified [2]. Compared to the sub-basal nerve fibers, the hyphae are slightly thicker, tend to branch at more obtuse angles, and their diameter tends to be the same across the entire length of the microorganism, ending in a rounded shape. The pathogen tends to invade the cornea both horizontally and vertically; therefore, the hyphae may be found at any depth, including the deep stroma. As the lesion advances, the center of the infiltrate appears as interlocking hyphae, masking other corneal structures, or linear structures with varying lengths interspersed throughout other corneal structures, such as nerves, epithelial cells, or stromal cells [18].

**Figure 7:**
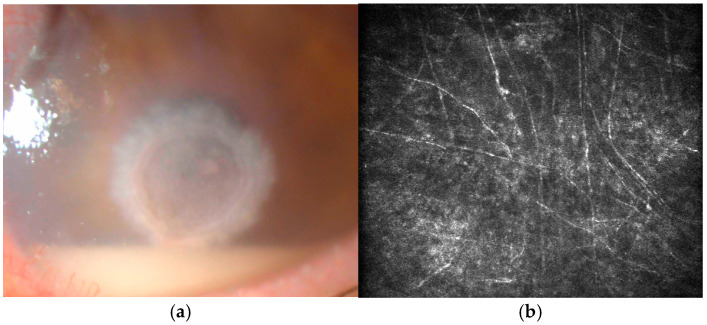
Fungal keratitis—Aspergillus. (**a**) A central white-gray feathery infiltrate with an overlying epithelial defect, hypopyon; (**b**) IVCM stromal slice—fungal hyphal network.

*Aspergillus* spp. hyphae appear as long structures (200–300 μm long, 3–5 μm wide). Reflectivity in Aspergillus hyphae tends to vary more than in Fusarium hyphae; they probably exhibit adventitious sporulation [2]. Brasnu et al. identified that Fusarium hyphae tend to branch at 90 degree angles [2]; however, in a study with a bigger cohort, little difference was discovered between the branching angles of Fusarium and Aspergillus, both averaging 60 degrees, and identifying the fungus using the angle of branching is currently considered an unreliable method [23]. A commonly found aspect in Aspergillus keratitis is illustrated in Figure 7.

**Table 1:** AS-OCT and IVCM features of different fungal species.

Fungus Type	AS-OCT Features	IVCM Features
*Candida* spp.	Hyperreflective homogenous focal lesions with well-defined borders; Epithelium overlying the lesion can be thinned or intact; Hyperreflective granules can sometimes be distinguished within the lesion; High reflectivity of the lesion can be a source of shadowing of the underlying tissue; Intensity of the inflammatory response is greater in bacterial infections [5,24].	Spores—round, highly reflective structures, 2–5 μm in diameter; Pseudohyphae (aggregated fungal cells)—elongated, beaded, linear structures (10–40 μm long, 5–10 μm wide); A higher density of dendritic and inflammatory cells [2,20,25].
*Fusarium* spp.	Extensive stromal hyperreflective infiltrates, often spanning the full thickness of the cornea; Ill-defined hyperreflective borders with surrounding stromal hyporeflective tissue; Cystic necrotic areas, which are seen as hyporeflective areas; Endothelial plaques and hypopyon [5,26].	Hyphae—brightly reflective, linear, branching, thin structures 200–300 μm long and 3–5 μm wide; A septated, slightly beaded aspect of the hyphae can be sometimes identified [2,23].
*Aspergillus* spp.	Findings in Aspergillus keratitis are similar to Fusarium; Early in the infection—localized stromal micro-abscesses appearing as small hyperreflective foci with surrounding low reflectivity; Progression—more diffuse stromal necrosis develops (coalescing large, cystic, low-reflective spaces within a hyperreflective stroma); Surrounding edema and any ring infiltrate will appear as thickened, hazy regions; Frequent endothelial plaques; Associated with hypopyon more frequently compared to Fusarium [5,26,27].	Septate fungal filaments appear very similar to Fusarium: slender (~5–12 μm wide) branching hyphae [24]; Reflectivity in Aspergillus hyphae tends to variate more than in Fusarium hyphae; probably exhibits adventitious sporulation [2].
Dematiaceous fungi (pigmented filamentous molds, e.g., Curvularia, Exserohilum, Alternaria)	Alternaria: corneal stromal appearance in Alternaria keratitis on AS-OCT is similar to other filamentous fungi—hyperreflective infiltrates with possible anterior stromal necrotic spaces—but the hallmark in advanced cases is the presence of free-floating, filamentous lesions in the anterior chamber [28].	Thick, septated hyphae, often larger in caliber and appearing as short, fragmented filaments with fewer branches; Curvularia hyphae are ~12 μm thick and relatively short (~30–40 μm length) with no obvious branching noted on IVCM; Exserohilum forms long septate hyphae (averaging ~320 μm length) about ~8 μm in width, with a broad branching angle; Alternaria with ~8 μm wide, ~130 μm long hyphae branching at ~40°; The pigmented fungi produce high-contrast hyphae on IVCM, but the filaments may appear more short and thick with fewer intertwining networks compared to Fusarium [24]

Abbreviations. IVCM = in vivo confocal microscopy, AS-OCT = anterior segment optical coherence tomography.

### 3.4. Anterior Segment Optical Coherence Tomography (AS-OCT)

AS-OCT allows for precise measurements of various parameters that can be used to assess the risk level of the infection: corneal thickness, the depth and width of corneal infiltrates, the presence of satellite lesions, endothelial plaques, or necrotic, cystic areas [26]. An overview of the most common AS-OCT findings is illustrated in Table 1.

Certain features can differentiate active from inactive lesions: active lesions present indistinct margins and posterior shadowing, while inactive lesions have clearly delimited margins [26]. Dynamically following the extent of the lesion during treatment can be used in assessing the efficacy of the antifungal treatment. The rate of decrease in corneal thickness may be an indication of the risk of perforation [26] and the need for therapeutic emergency keratoplasty.

*Candida* spp. lesions are seen as hyperreflective, homogenous, focal lesions with well-defined borders, similar to infiltrates in bacterial infections. The epithelium overlying the lesion can be thinned or intact, and can involve the graft interface and posterior stroma in previously transplanted eyes. AS-OCT shows a dense hyperreflective deposit at the graft–host interface or along the endothelium. Hyperreflective granules can sometimes be distinguished within the lesion. The high reflectivity of the lesion can be a source of shadowing of the underlying tissue. The boundaries of the endothelial plaques, when they do appear, cannot be distinguished from the stroma. While a degree of thickening is visible in the surrounding stroma, the intensity of the inflammatory response is greater in bacterial infections [5,24].

*Fusarium* spp. typically affect the anterior to mid-stroma, with severe cases progressing towards the Descemet’s membrane and perforation. The infiltrates present feathery borders, stromal edema—visible on AS-OCT as ill-defined hyperreflective borders with surrounding stromal hyporeflective tissue—and cystic necrotic areas, which are seen as hyporeflective. Endothelial plaques and hypopyon are common findings [5].

*Aspergillus* spp. lesions tend to also have feathery margins, but the surface tends to be more elevated compared to *Fusarium* spp. OCT aspects show uneven hyperreflectivity with interspersed hyporeflective pockets of necrosis, usually with extensive involvement of the stroma. Surrounding edema and any ring infiltrate will appear as thickened, hazy regions. Cases tend to develop endothelial plaques and be associated with hypopyon more frequently compared to Fusarium. The infiltrate is seen as a hyperreflective lesion spanning the full thickness of the stroma, due to its tendency to be more invasive and develop in a full-thickness stromal infiltrate quicker than Fusarium. Stromal necrosis can be seen as low-reflectivity areas distributed throughout bright areas. Altered stromal architecture with stromal melting is more common. In practice, the posterior Fusarium/Aspergillus exudates blend into underlying layers, which on OCT appear as an irregular, poorly defined border (unlike the sharp line seen in bacterial keratitis plaques) [5]. 

### 3.5. Medical and Surgical Management

The cornerstone of the management of fungal keratitis is represented by medical antifungal therapy. Topical therapy can include Natamycin 5% as the first-line topical treatment, particularly effective against filamentous fungi [15,29]; Voriconazole 1%, with broad-spectrum antifungal efficacy via ergosterol synthesis inhibition, is beneficial in cases involving filamentous fungi and yeasts, or when initial treatment fails [15,30].

Amphotericin B 0.15% is specifically indicated for Candida keratitis, but lacks efficacy against Fusarium species; it is thus reserved for natamycin-resistant cases or yeast infections [15,31]. Initial adjunctive antiseptic treatment with chlorhexidine or povidone-iodine may help reduce fungal load and aid in the treatment of multi-resistant infections [32,33].

Systemic antifungals such as voriconazole, itraconazole, fluconazole, ketoconazole, and miconazole are indicated for extensive infections involving limbal or scleral extension, or endophthalmitis [15]. Due to the significant incidence of bacterial coinfections, particularly in yeast-associated keratitis, broad-spectrum topical antibiotics (e.g., fluoroquinolones) are frequently administered [10,11,34].

Emerging adjunctive treatments, including photoactivated chromophore for keratitis-corneal crosslinking (PACK-CXL) and photodynamic therapy (PDT), have demonstrated preliminary promise; however, their definitive therapeutic role requires further investigation [12,35].

The antifungal agents (e.g., amphotericin B or voriconazole) may be administered directly into the corneal stroma or anterior chamber or used as a washout for the stromal interface, in cases of lamellar keratoplasty [32].

Indications for performing therapeutic keratoplasty relate to the nature and aggressiveness of the fungal agent, depth extension, perforation risk and visual axis involvement. When considering therapeutic keratoplasty, if the patient meets the criteria, lamellar keratoplasty can be a good option due to lower rates of rejection and a lower need for steroid treatment after the procedure [15]. Some general guidelines for each type of therapeutic keratoplasty have been proposed in the literature; however, the particularities of each case must always be taken into consideration. Several indications have been proposed in the literature, using criteria seen on AS-OCT and IVCM. The recipient peripheral cornea should ideally include 1–1.5 mm of clear cornea, with absent fungal infection [15]. It is reported that half of all fungal keratitis cases need therapeutic keratoplasty [6,20].

T-DALK indications include the depth of the infiltrate involving only the anterior 150–300 µm of stroma, no Descemet membrane involvement, the lesion being confined in the central 6 mm optical zone, the absence of hypopyon, or endothelial plaques [36,37]. Both filamentous fungi and yeasts can meet the criteria, however more aggressive species with a more rapid stromal penetration are less likely to meet the criteria.

TPK is indicated by the following: a corneal perforation or impending risk of perforation; descemetocele formation; a mean infiltrate diameter of >6 mm [6,15]; if deep necrosis is present, seen on AS-OCT as hyporeflective cystic spaces within a hyperreflective infiltrate; posterior spread; or endothelial plaques, seen as indistinct, irregular, hyperreflective material extending across the endothelium–anterior chamber interface [5,38]. IVCM findings indicating the need for TPK are dense, confluent networks of fungal filaments in the posterior one-third of the stroma or closer to the Descemet’s membrane and branching hyphae after one week of maximal antifungal treatment [39].

An important complication following corneal transplantation in fungal keratitis is the recurrence of the infection. Retrospective studies have reported that approximately 6–7% of cases suffer from recurrence [36,40]. Risk factors include preoperative hypopyon, the infiltrate involving the limbus, corneal perforation, lens infection, topical corticosteroids, a preoperative history of fungal keratitis, and the storage of the donor cornea without antifungal agents. Most commonly, the causative agent is *Candida* spp. [40,41]. Interestingly, the recurrence rate was not significantly different between penetrating and lamellar keratoplasty [36,40]; however, a better prognosis was found significantly more often in patients with recurrences following lamellar keratoplasty [36].

Our cases illustrate the usefulness of IVCM and AS-OCT in management from the first consultation of the patient to several years after transplantation, from identifying the etiological agents more quickly to monitoring the treatment response dynamically. The first case involves a primoinfection with a strain of Candida that quickly developed resistance to fluconazole, followed by a recurrence of the infection in the graft, also with Candida. IVCM revealed unexpected Acanthamoeba cysts and trophozoites; however, the cyst and trophozoite count was low and the infection was confined to the anterior and mid-stroma. The relatively rare finding of a mixed fungal and Acathamoeba coinfection in corneal scrapings [41,42] also underlines the invaluable insights of IVCM. Recent studies suggest that fungi–Acanthamoeba coinfection could be more common than previously thought [43,44]. We adjusted the medical treatment to also include the povidone-iodine wash 2%, shown to also have cysticidal action [45,46,47]. In our experience, povidone-iodine 2% wash has improved outcomes both in fungal infections and in Acanthamoeba keratitis.

The extent of the infiltrate was evaluated with AS-OCT—at the primoinfection, the infiltrate was confined to the anterior 300 μm of the stroma. After the recurrence of the infection, the infiltrate involved almost the whole thickness of the donor corneal graft, but with the host posterior stroma and Descemet membrane intact. Due to the tendency of the infiltrate to remain stationary, we decided to perform a re-DALK 10 days after admission, to try to limit the extension in the host corneal tissue.

We monitored the response to treatment by using AS-OCT to measure the modifications in the size and reflectivity of the infiltrate. Initiating the voriconazole treatment and interface washout seemed successful at first, but IVCM showed an increase in fungal pseudohyphal network density after 10 days of treatment. This evidence of treatment resistance was key in the timing of the second DALK. The absence of fungal lesions in the deep stroma was observed at every follow-up IVCM.

The second case illustrates a very aggressive filamentous fungal infection, which extended very quickly towards the anterior chamber, significantly raising the risk of the fungal colonization of inaccessible, intraocular structures that might be a starting point for infection recurrence at a later time. The key was quick admission and therapeutic penetrating keratoplasty, with very frequent follow-ups due to the high risk of rejection. Withholding corticotherapy was also a complicating factor as intervening in an eye with an active infection also increases the rejection risk.

As recurrence risk is very high regardless of the type of keratoplasty performed, especially when the infection reaches the anterior chamber [48], IVCM could play a role in a quick in vivo examination of any suspicious lesions that might appear during the postoperative period.

It is important to note that IVCM and AS-OCT both have a series of limitations. IVCM images a very small field at one time, making targeting the same spot almost impossible; therefore, it is more suited for a qualitative evaluation of the changes, such as the presence of a certain pathogen and its characteristics. Some attempts to standardize the acquisition have been made, with variable degrees of success [49]. While AS-OCT does not have sufficient resolution to detect characteristics such as cysts or fungal hyphae, it offers information on the gross morphology of the infiltrate (extension, size, and homogeneity of reflectivity). The HRT-3 RoStock Cornea module provides depth information during the acquisition, which could, correlated with AS-OCT depth data, enhance the planning of the IVCM acquisition area.

A promising further direction, given the wide variability of clinical aspects in fungal keratitis, is the research into identifying possible pathognomonic IVCM and AS-OCT characteristics in different types of fungi, as well as how these aspects change in response to treatment. The recent advances in deep learning and artificial intelligence could be used for a variety of applications. Multiple groups have demonstrated the usefulness of artificial intelligence in identifying Acathamoeba [50,51] and fungi [52,53,54]. Further directions could include examining possible pathognomonic characteristics and identifying pathogen-specific patterns, identifying the rate of progression, aggressivity of a pathogen, and the risk for perforation.

## 4. Conclusions

Achieving optimal results in complex fungal keratitis cases is a challenge that requires very close and frequent monitoring. High-resolution corneal imaging, specifically AS-OCT and IVCM used in tandem, could provide a step forward in identifying pathognomonic patterns for different etiological agents, and capture aspects that might provide valuable insights in understanding both corneal responses to infectious injury and which aspects represent a good response to medical treatment. The timing of the surgical treatment for better outcomes in aggressive infectious keratitis could also be improved by correlating the results of the two imaging techniques.

## Data Availability

No new data were created or analyzed in this study. Data sharing is not applicable to this article.

## Abbreviations

The following abbreviations are used in this manuscript:

IVCM	in vivo confocal microscopy
AS-OCT	Anterior Segment Optical Coherence Tomography
DALK	Deep Anterior Lamellar Keratoplasty
PK	Penetrating keratoplasty
TPK	Therapeutic penetrating keratoplasty
T-DALK	Therapeutic Deep Anterior Lamellar Keratoplasty
PKIK	post keratoplasty infectious keratitis
